# Transcriptomics and starch biosynthesis analysis in leaves and developing seeds of mung bean provide a basis for genetic engineering of starch composition and seed quality

**DOI:** 10.3389/fpls.2024.1332150

**Published:** 2024-05-01

**Authors:** Kamolchanok Umnajkitikorn, Pakpoom Boonchuen, Rattanavalee Senavongse, Sunanta Tongta, Yu Tian, Yaqi Hu, Bent Larsen Petersen, Andreas Blennow

**Affiliations:** ^1^ School of Crop Production Technology, Institute of Agricultural Technology, Suranaree University of Technology, Nakhon Ratchasima, Thailand; ^2^ School of Biotechnology, Institute of Agricultural Technology, Suranaree University of Technology, Nakhon Ratchasima, Thailand; ^3^ School of Food Technology, Institute of Agricultural Technology, Suranaree University of Technology, Nakhon Ratchasima, Thailand; ^4^ Department of Plant and Environmental Sciences, Copenhagen University, Frederiksberg, Denmark

**Keywords:** mung bean, starch, seed development, transcriptome, precise genetic engineering, starch biosynthesis

## Abstract

Mung bean starch is distinguished by its exceptional high amylose content and regulation of starch biosynthesis in leaves and storage tissues, such as seeds, share considerable similarities. Genetic engineering of starch composition and content, requires detailed knowledge of starch biosynthetic gene expression and enzymatic regulation. In this study we applied detailed transcriptomic analyses to unravel the global differential gene expression patterns in mung bean leaves and in seeds during various stages of development. The objective was to identify candidate genes and regulatory mechanisms that may enable generation of desirable seed qualities through the use of genetic engineering. Notable differences in gene expression, in particular low expression of the Protein Targeting to Starch (PTST), starch synthase (SS) 3, and starch branching enzyme1 (SBE1) encoding genes in developing seeds as compared to leaves were evident. These differences were related to starch molecular structures and granule morphologies. Specifically, the starch molecular size distribution at different stages of seed development correlated with the starch biosynthesis gene expression of the SBE1, SS1, granule-bound starch synthases (GBSS) and isoamylase 1 (ISA1) encoding genes. Furthermore, putative hormonal and redox controlled regulation were observed, which may be explained by abscisic acid (ABA) and indole-3-acetic acid (IAA) induced signal transduction, and redox regulation of ferredoxins and thioredoxins, respectively. The morphology of starch granules in leaves and developing seeds were clearly distinguishable and could be correlated to differential expression of SS1. Here, we present a first comprehensive transcriptomic dataset of developing mung bean seeds, and combined these findings may enable generation of genetic engineering strategies of for example starch biosynthetic genes for increasing starch levels in seeds and constitute a valuable toolkit for improving mung bean seed quality.

## 1 Introduction

Mung bean is a legume crop that has been used for human consumption for millennia. Mung bean, in the form of seeds, starch and sprouts, is an inexpensive vital source of protein, carbohydrate, minerals and dietary fibers (Campos-Vega et al., 2010; Rebello et al., 2014). Mung beans contain 22.9-23.6% protein, 1.2% oil, and 58.2−61.8% carbohydrate (Pehrsson et al., 2013; Masari et al., 2017; Tayade et al., 2019). Due to its short-life cycle and nutrient composition, mung bean is a cash crop or rotational crop with other main crops such as rice and corn in many countries, including China, India and Thailand (Chadha, 2010).

A number of studies have consistently demonstrated that mung bean starch, along with starch of other pulses, possesses higher amylose levels in comparison to starches in cereals, or in tubers of for example potato (Kim et al., 2007; Huong et al., 2021). Mung bean starch constitutes approximately 26 to 31% of the seed, and is important in the production of transparent noodles due to its distinctive qualities, including high shear resistance, limited swelling, and excellent granular stability (Oates, 1991). Presently, there is an increasing focus on health and well-being among consumers. Resistant starch, being a functional food component in a number of starch-rich diets, offers health benefits, such as potentially reduced blood glucose levels and exhibits prebiotic effects in the colon (Goldring, 2004). Owing to their high protein and resistant starch content, mung beans hold promising potential in the functional and health-oriented food market. However, molecular mechanisms involved in seed development and starch biosynthesis are relatively unknown and generation of for example additional transcriptomic data may enable modulation of starch composition and yield, through overexpression or knock out, or combinations hereof, of starch biosynthetic or regulatory genes.

Starch is synthesized in the plastids, and is essential in plant growth and development. Starch in leaves is synthesized and accumulates within chloroplasts during daylight hours and is subsequently being broken down during the night to sustain energy for nocturnal growth and development. Leaf-based starch is notably susceptible to degradation, whereas starch stored in organs like seeds, storage roots, or tubers exhibits greater metabolic stability. Storage starch serves as an energy source during germination. Biosynthesis of starch in both leaves and seeds occurs within plastid stroma and involves both similar and distinct enzymatic processes and regulatory mechanisms, typically facilitated by genetically distinct isoforms (Nakamura, 2015).

The process of starch biosynthesis is highly conserved across plant species, although the number of homologous enzymes involved can vary. This variability is influenced by factors such as tissue specificity and the degree of redundancy within the biosynthetic pathway (Tanackovic et al., 2014). It is important to note that starch biosynthesis and metabolism may be regulated differently depending on the specific organ and species under consideration. This differential regulation allows for fine-tuning of starch production to meet the specific needs of different plant organs and species. Additionally, these variations in starch biosynthesis enzymes and regulation have significant implications for plant development and crop domestication, as they contribute to the diverse range of starch compositions observed across different plant species and varieties (Tetlow and Emes).

We therefore aim to investigate the gene expression profile across the developmental stages of mung bean seed, compared with leaf. Even though most enzymes in starch biosynthesis have been identified, and genetic engineering of these has resulted in various starch compositions and functionalities, genetic engineering of starch biosynthesis may confer growth retardation and yield penalties (Smith, 2008; Blennow, 2018). For example, silencing of starch branching enzyme (SBE) causes growth retardation in barley during germination and as young plants with 22% yield penalty due to a decrease in spike number, grains per spike and grain weight (Carciofi et al., 2012). DNA-free CRISPR/Cas mediated genome editing of SBE1 and SBE2 in potato, generated some lines containing multiple mutations in both genes, which exhibited growth retardation with a significant reduction in both size and total tuber yield, ranging from 60% to 80% when compared to the parental variety (Zhao et al., 2021).

In *Nicotiana benthamiana*, transient silencing of plastidial soluble inorganic pyrophosphatase (psPPase), regulating the pyrophosphate hydrolyze during the initial step of starch biosynthesis, lead to reduced carbon assimilation, decreased starch content, reduced chlorophyll and carotenoid biosynthesis, and ultimately reduced growth (George et al., 2010). Not all starch biosynthetic gene silencing’s or knockouts result in growth retardation; for example, RNAi silencing of starch branching enzymes of class II (SBEIIa) in durum wheat (Sestili et al., 2010). Starch synthase III (SS3) mutant in Arabidopsis displayed altered leaf structure and excess starch accumulation at the end of light period but did not display a significant growth phenotype, despite incomplete starch degradation during the night (Zhang et al., 2005).

Impaired function, and depending on species, of some starch biosynthetic genes contributes to altered growth and development.In tobacco, knockout mutants of the plastidial phosphoglucomutase (PGM), which converts fructose 6-phosphate to Glc1P after the catalytic reaction of phosphoglucoisomerase (PGI), show significant growth retardation, which, however, is not found in mutants of pea and *Lotus japonicus* (Stitt and Zeeman, 2012). In contrast, loss-of-function mutants of the glucan water-dikinase 1 (GWD1) in *L. japonicus* has a strong starch-excess phenotype and severe growth defects (Stitt and Zeeman, 2012), while downregulation of GWD1 in rice (Wang et al., 2021), or potato (Viksø-Nielsen et al., 2001) has no effect on growth (Wang et al., 2021), with no explicit rational to these apparent differences.

The sequential expression of these genes during seed development, compared to the expression in leaf may provide clues to interactions between starch biosynthetic enzymes and their interactions with their substrate, including their potential for growth promotion or detrimental effects in genetic engineering settings. Here, we performed a comparative transcriptomic analysis of a number of key starch biosynthetic target genes in different stages of seed development and leaf, which in future genome editing or other breeding strategy settings, may enable choosing target genes that also include minimizing the risk of growth retardation, resulting from concomitant starch modification in leaves.

## 2 Materials and methods

### 2.1 Plant materials

Economically relevant cultivars of Mung bean (*Vigna radiata*), regularly grown in Thailand: Chai Nat 3 (CN3), Chai Nat 36 (CN36), Chai Nat 72 (CN72), Chai Nat 84-1 (CN84-1), Kam Pang Saen 1 (KPS1) and Suranaree 1 (SUT1), was grown in the field at Suranaree University of Thailand.

### 2.2 Starch, amylose and resistant starch content of Thai commercial cultivars

#### 2.2.1 Mung bean starch extraction

Mung bean seeds (150g) were immersed in distilled water (750 mL) in a beaker and heated at 50°C for 3 h. The seed coats were removed. The dehulled mung bean seeds were ground and prepared to be a slurry which was suspended in 0.2% sodium hydroxide (450 mL). After steeping overnight, the extracted starch was filtered through a 140-mesh sieve, washed and centrifuged. The starch was dried at room temperature and gently ground to powder (Duyen et al., 2020).

#### 2.2.2 Resistant starch analysis

A modified Association of Official Agricultural Chemists (AOAC) method 2002.02 was applied for RS determination. Each sample (100 mg) was digested by addition of 4 ml of enzyme reagent (30 U/ml of pancreatic α-amylase and 3U/ml of amyloglucosidase) and incubated at 37°C for 16 h. The reaction was terminated by adding 4 mL of ethanol (99%), mixed and centrifuged. The supernatant was discarded and the remaining starch (pellet) was re-suspended and centrifuged two times in 50% ethanol (v/v). The RS fraction was solubilized by addition of 2 mL of 2 M KOH, hydrolyzed with amyloglucosidase. The liberated glucose was quantified using glucose oxidase/peroxidase reagent (GOPOD was purchased from Megazyme Co. Ltd Wicklow, Ireland) and converted to the RS content.

#### 2.2.3 Determination of amylose content

Starch samples were dissolved in 5mL of 1M NaOH (10 mg/mL) and gelatinized in a boiling water bath. The gelatinized sample (5mL) was transferred into a volumetric flask, followed by addition of 70 mL of DI water, 1 mL of 1M acetic acid and 2 mL of iodine solution (0.2 g of iodine and 2.0 g of potassium iodide in 100 mL of aqueous solution). The mixture was adjusted with DI water to 100 mL. The absorbance at 610 nm was monitored against blank (without starch sample). The amylose content was calculated from calibration curves of amylose-amylopectin mixtures with known content (Juliano, 1971).

#### 2.2.4 Iodine staining and photography

Mung bean seeds stage 1, 2, 3, 4, 5 and mature seeds were free-hand cross- and long-sectioned using a razor blade on petri dish glass. The sections were stained with iodine solution (15 mM I_2_, 0.1 M potassium iodide) solution and left for 2-3 minutes. The stained sections were examined under a Leica Microsystems 10447197 EZ4 Stereo Microscope (Leica Nanterre, France).

### 2.3 RNA extraction and transcriptome analysis

The flowers were tagged on the day they had fully opened. Seeds from each developmental stage were collected as follows: stage 1 included seeds at 5**-**6 days after flowering (DAF); stage 2 comprised seeds at 10**-**11 DAF; stage 3 involved seeds at 14**-**15 DAF; stage 4 encompassed seeds at 17**-**18 DAF; stage 5 consisted of seeds at 24**-**25 DAF; and mature seeds were those at more than 30 DAF. All pod tissues were removed, and seeds were chopped into small pieces, then immediately frozen in liquid nitrogen. The youngest fully expanded leaf was collected during stages 1**-**2 of seed development and immediately frozen in liquid nitrogen. Tissues were subsequently ground into powder using liquid nitrogen, a mortar, and pestle before RNA extraction. The samples are stored at -80°C until used. Due to the high content of starch in samples, total RNA was extracted according to a modified protocol of Vennapusa et al. (2020) with some slight modifications. Concisely, a total of 100 mg ground tissue, which was pulverized in liquid nitrogen, was added with 600 µl of RNA extraction buffer. Acid-Phenol: Chloroform, pH 4.5: isoamyl alcohol, 125:24:1) (Invitrogen) and chloroform were used to extract total RNA. The aqueous phase was collected, LiCl (2 M final concentration) and sodium acetate (0.3 M final concentration) were added and the total RNA was precipitated by incubating overnight at -20°C, following by centrifugation at 12,100 g. The pellets were pooled, washed by 1 ml 2 M LiCl, followed by pre-chilled 70% ethanol at RT. The integrity and quantity of purified RNA were examined by 1% agarose gel electrophoresis and Nanodrop 2000 Spectrophotometer (Thermo Scientific), respectively.

RNA libraries were prepared using 5 μg of total RNA extracted from both seeds and leaves. Sequencing and subsequent bioinformatics analysis were conducted by GENEWIZ in Suzhou, China. Briefly, mRNA was initially enriched from the total RNA, followed by adapter ligation. Afterward, size selection of adaptor-ligated DNA fragments was performed. Libraries featuring various indexes were pooled together and loaded onto an Illumina HiSeq instrument for sequencing, employing a 2 × 150 paired-end (PE) configuration in accordance with the manufacturer’s instructions.

The raw data were subjected to preprocessing using Cutadapt version 1.9.1 (Martin, 2011). This preprocessing involved the removal of adapter sequences, as well as bases containing ‘N’ at either the 5’ or 3’ ends, and the trimming of reads that were shorter than 75 base pairs. Subsequently, the resulting sequences were aligned to the reference genome (GCA_000741045.2) using BWA version 0.7.5a-r405 (Li and Durbin, 2009). The raw data were available in NCBI under the SRA accession PRJNA1033708. Differential expression analysis was carried out, and genes meeting the criteria of Padj < 0.05 and log2|fold change| > 1 were designated as differentially expressed genes (DEGs) for further investigation.

Enrichment analyses for Gene Ontology (GO) and Kyoto Encyclopedia of Genes and Genomes (KEGG) of the differentially expressed genes (DEGs) were conducted using the clusterProfiler R package, which includes correction for gene length bias (Yu et al., 2012). GO terms or KEGG pathways with adjusted P-values below 0.05 were deemed significantly enriched within the DEG dataset.

### 2.4 Gene expression analysis by qRT-PCR

To assess and validate the differential expression profiles as identified through RNA-seq analysis, quantitative real-time polymerase chain reaction (qRT-PCR) was employed to examine the expression of differentially expressed genes (DEGs) at various stages of seed development and in leaves. For this purpose, 1 µg of total RNA was utilized to generate complementary DNA (cDNA) using the iScript cDNA Synthesis Kit (Bio-rad, USA). Subsequently, qRT-PCR analysis was conducted, employing specific primers (**Supplementary Table S1**) and the Luna Universal qPCR Master Mix (New England Biolabs, USA). The relative expression levels were determined using the 2^-ΔΔCt^ method (Livak and Schmittgen, 2001) and were normalized to beta-actin. Each experiment was carried out in triplicate.

### 2.5 Starch extraction from leaf and developing seed

The powder of leaves and seeds at different stages were freeze-dried and starch extracted with the modified method from Hostettler et al. (2011). Dried leaf or seed powder was mixed with 0.05M NaOH and mixed for 1 h (in 1:10 ratio), sieved through the 35/38 µm-filter, collected the pellet with centrifugation at 3000 g, and then added HEPES-Tritox buffer (20 mM HEPES-KOH, 0.2 mM EDTA, 0.5%Triton X-100 pH 8.0). The slurry was stirred for 1 h, centrifuged at 3000 g, washed with milliQ water twice and then washed with 96% ethanol to remove any remaining pigment. The pellet was washed with milliQ again before freeze-drying the samples. The starch powder from leaves and the 5 developmental stages was kept at room temperature in a closed container until use.

### 2.6 Microscopic analyses of starch

The morphology of starch granules was investigated by mounting them flat on a grid, sputter-coated with gold. and monitored using Field Emission Scanning Electron Microscopy (FE-SEM) (FEI Quanta 200) at the University of Copenhagen, Panum campus, Core Facility for Integrated Microscopy. Granular morphology was observed and recorded at 5000x, 10000x, 25000x magnification.

### 2.7 Molecular size distribution

The size distributions of both native (fully branched) starch molecules and starch chains were assessed according to the method outlined by Li et al. (2016), with some minor adjustments. This analysis utilized size exclusion chromatography with a triple detector array (SEC-TDA) (Viscotek in Malvern, UK). The system was equipped with GRAM 1000 SEC columns (Polymer Standards Service GmbH in Mainz, Germany), and connected to a refractive index detector (PN3140) (PostNova Analytics in Landsberg, Germany).

For the analysis of native starch, 5 mg of the samples were dissolved in 1 mL of DMSO/LiBr solution (0.5% w/w, obtained from Avantor, US) and then incubated at 80°C overnight, centrifuged at 20,000 g for 5 minutes. The resulting supernatant was injected into the SEC system. Elution was carried out using DMSO/LiBr solution (0.5%) at a flow rate of 0.5 mL/min, with the column temperature set to 65°C (Tian et al., 2024).

Debranched samples were prepared by heating starch dispersions in screw-cap tubes with DMSO/LiBr (5 mg/mL) at 80°C for 3 hours. The gelatinized starch was collected after centrifugation at 4000g for 10 minutes, using absolute ethanol, and then allowed to dry in an open-air environment at ambient temperature. Further debranching was accomplished by adding 4 μL of isoamylase (Megazyme, E-ISAMY, 200 U/mL) and incubating in 1 mL of sodium acetate buffer (0.01M, pH 4.0) at 40°C for 3 hours, followed by freeze-drying. Prior to injection into the SEC system, the debranched samples (5 mg/mL) were dissolved in DMSO/LiBr (Tian et al., 2024).

### 2.8 Statistical analysis

The statistical analysis of mean values and their associated standard errors was carried out using SPSS software (Version 16.0 for Windows; SPSS Inc., Chicago, IL, USA). To compare means, Duncan’s multiple range test and the independent sample t-test were applied (for enzyme activity and gene expression data), with a significance level set at p < 0.05. Specific details regarding sample sizes and replications can be found in the respective figure and table legends.

## 3 Results

### 3.1 Starch, amylose and resistant starch content

Six economically important mung bean cultivars were first analyzed for total starch content, amylose content, and resistant starch content in order to select a suitable cultivar for further starch biosynthetic gene expression analysis. The tested mung bean seeds contained 37-43% total starch, 31-33% of amylose, and 7-8% of resistant starch in the mature seeds (**Supplementary Figure 1**). CN84-1 contained the highest starch percentage (**Supplementary Figure 1A**), and the total amylose content was highest in CN3, CN72 and KPS1 (**Supplementary Figure 1B**). While there was no significant difference in resistant starch content among the 5 cultivars: CN3, CN36, CN72, CN84-1 and KPS1, SUT1 had a 1-1.5% lower resistant starch content (**Supplementary Figure 1C**). Based on this analyzed composition, CN84-1 was selected for the further analyses performed in this study.

**Figure 1:**
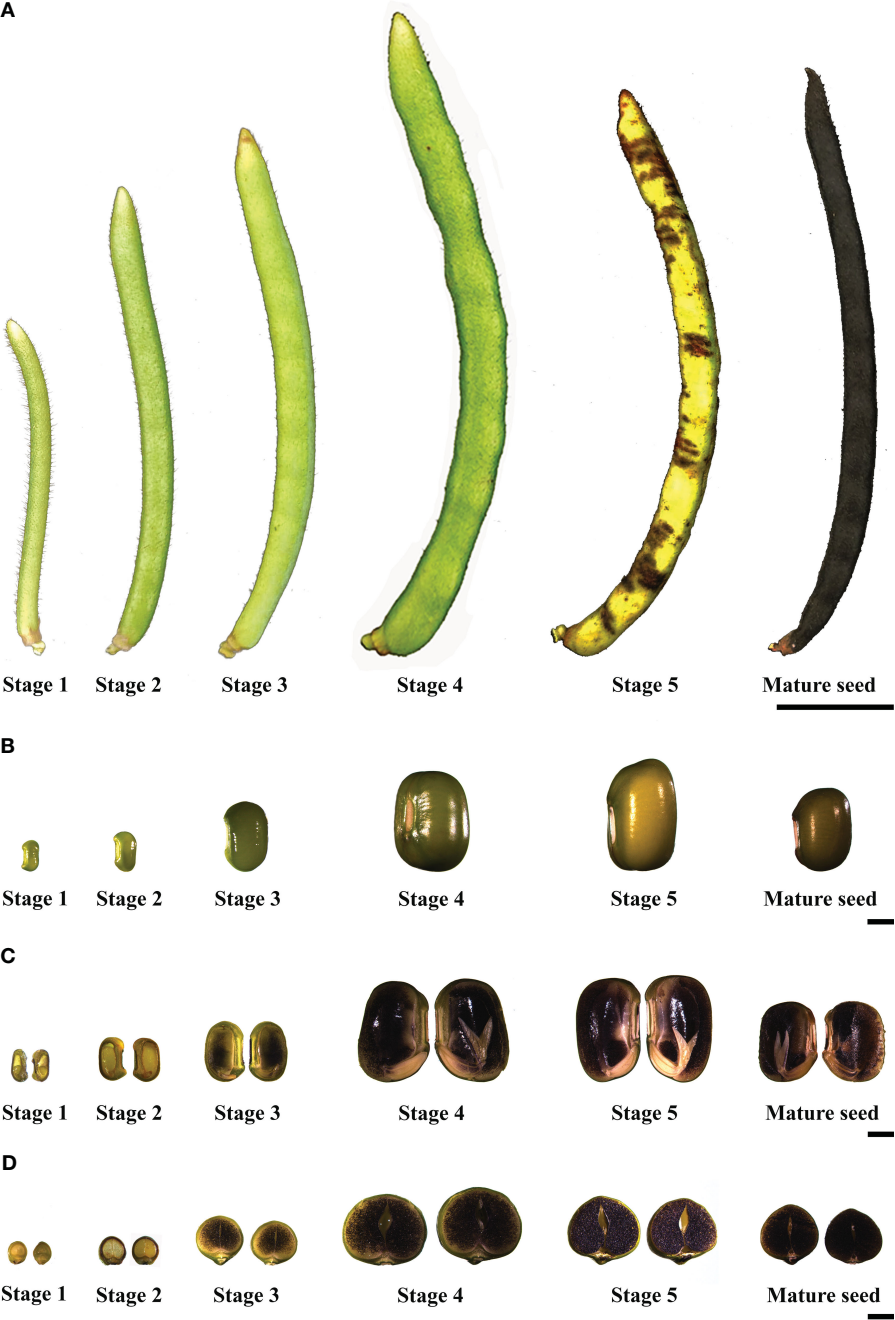
Different developmental stages of mung bean pods and seeds **(A)** Mung bean pods, the scale bar shows 5 mm; **(B)** Mung bean seeds, the scale bar shows 2 mm; **(C)** Long-section of mung bean seeds with iodine staining, the scale bar shows 2 mm; **(D)** Cross-section of mung bean seeds with iodine staining, the scale bar shows 2 mm. The developmental stages are as follows: Stage 1: seeds at 5-6 days after flowering (DAF). Stage 2: seeds at 10-11 DAF. Stage 3: seeds at 14-15 DAF. Stage 4: seeds at 17-18 DAF. Stage 5: seeds at 24-25 DAF. Mature seed: seeds at more than 30 DAF.

Moreover, iodine staining of the seeds in different stages illustrated that the starch in seed stage 1 and 2 preferentially accumulated in the seed coat with some accumulation in the cotyledons (**Figures 1C, D**). From stage 3, starch was predominantly accumulated in the cotyledon, and not the seed coat (**Figures 1C, D**).

### 3.2 Differentially expressed genes of the different stages of developing seed, compared to leaves

**Figure 2:**
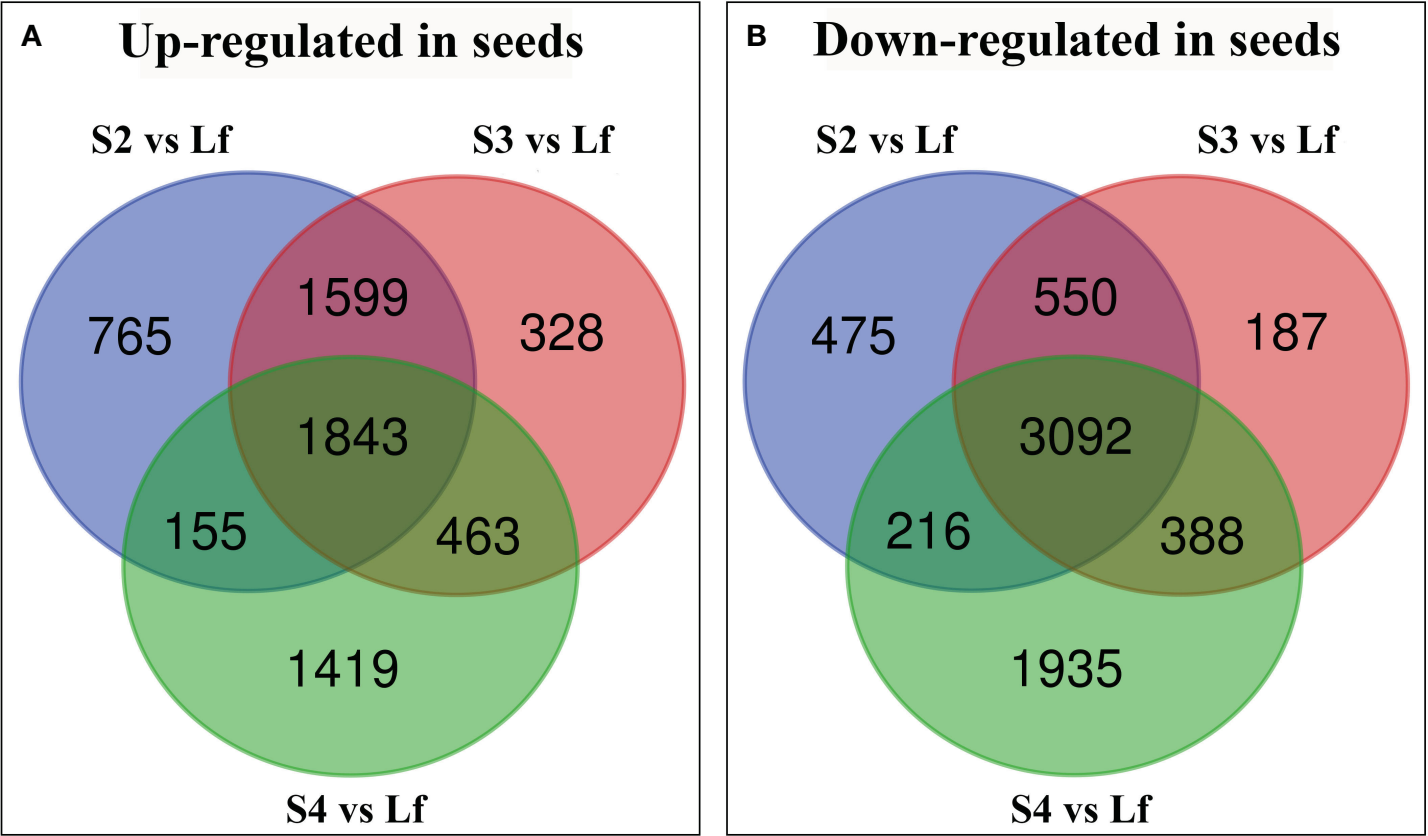
Venn diagram showing the number of unique differentially expressed genes (DEGs) in each treatment group of *Vigna radiata* cv. CN84-1: seed stage 2 compared with leaf (S2 vs Lf), seed stage 3 compared with leaf (S3 vs Lf), and seed stage 4 compared with leaf (S4 vs Lf). **(A)** the up-regulated gene in seeds **(B)** the down-regulated gene in seeds. Lf (Leaf).

To distinguish abundantly expressed genes in seeds or leaves, the differentially expressed genes (DEGs) of each developmental stage were statistically analyzed over the expression of the same genes in leaf tissue and the significantly different expressed genes were divided into 2 groups: genes that were up-regulated in developing seeds, and the genes that were down-regulated in seeds. The number of DEGs (13415) was significantly different in developing seeds and displayed an approximate 2-fold change (P value ≤ 0.05) with 6572 up-regulated and 6843 down-regulated (**Figure 2**). 1843 genes were up-regulated in all 3 stages of seed development while stage 4 displayed the highest number of up-regulated genes in seeds, when compared to leaves (**Figure 2A**). On the other hand, 3092 genes were down-regulated in seeds over all 3 stages of the developing seeds (**Figure 2B**). The developing seeds stage 4 displayed the differentially highest gene expression when compared to the other 2 stages (**Figures 2A, B**). At the same time, the stage 3 possessed the smallest number of genes that were distinctively up- or down-regulated, when compared to the other 2 stages (**Figures 2A, B**).

**Figure 3:**
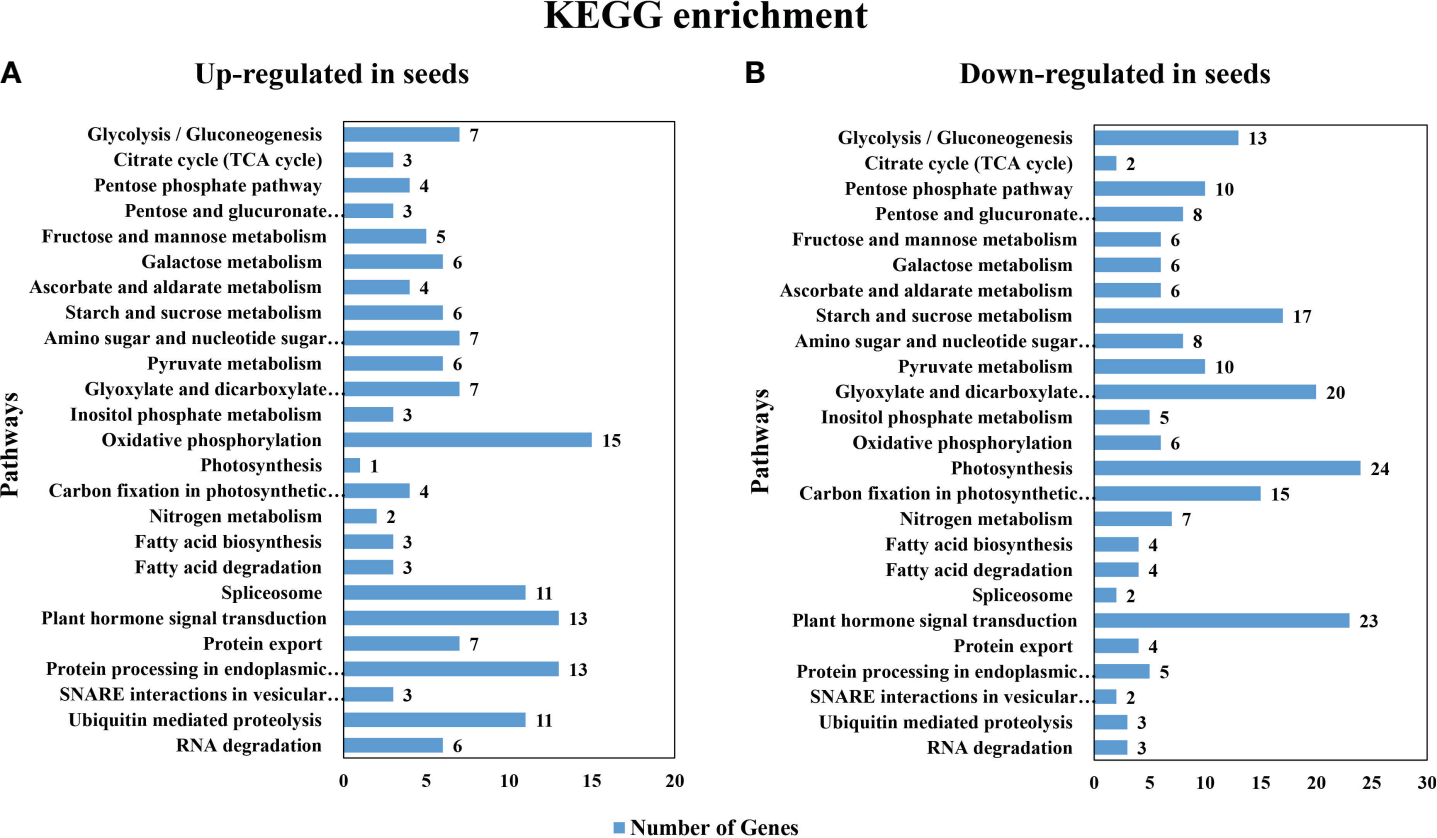
Gene Ontology (GO) enrichment of the most significant differentially expressed genes pathways in KEGG gene ontology **(A)** the up-regulated gene in seeds **(B)** the down-regulated gene in seeds.

The common up- or down-regulated genes at all 3 stages (1843 and 3092 genes, respectively as shown in **Supplementary Data Sheet 1**) were used as the inputs for pathway enrichment analysis using Kyoto Encyclopedia of Genes and Genomes (KEGG) analysis. The first 25 pathways with the highest number in DEGs that were enriched in each group included starch and sucrose metabolism pathways (**Figure 3**); however, with less up-regulated (6) than the down-regulated genes (17) (**Figures 3A, B**). Among these DEGs, β-fructofuranosidase [EC:3.2.1.26], β-glucosidase [EC:3.2.1.21], plastidial starch phosphorylase [EC:2.4.1.1], trehalose 6-phosphate synthase/phosphatase [EC:2.4.1.15/3.1.3.12] were found in both the up- and the down-regulated genes in seeds. On the other hand, only fructokinase [EC:2.7.1.4] and sucrose synthase [EC:2.4.1.13] were found to be up-regulated in seeds, while maltase-glucoamylase [EC:3.2.1.20 3.2.1.3], endoglucanase [EC:3.2.1.4], sucrose-phosphate synthase [EC:2.4.1.14], glucose-6-phosphate isomerase [EC:5.3.1.9], ectonucleotide pyrophosphatase/phosphodiesterase family member 1/3 [EC:3.1.4.1 3.6.1.9], glucose-1-phosphate adenylyltransferase [EC:2.7.7.27], starch synthase [EC:2.4.1.21], granule-bound starch synthase [EC:2.4.1.242], α-α-trehalase [EC:3.2.1.28], α-amylase [EC:3.2.1.1], β-amylase [EC:3.2.1.2] were down-regulated in seeds. Moreover, the most abundant KEGG pathways that were up-regulated in seeds were oxidative phosphorylation, plant hormone signal transduction and protein processing in endodermic reticulum (**Figure 3A**). In contrast, the down-regulated genes in seeds belonged primarily to pathways of photosynthesis, plant hormone signal transduction and the glyoxylate and dicarboxylate metabolism (**Figure 3B**).

### 3.3 Differentially expressed genes of the starch biosynthesis pathway

**Figure 4:**
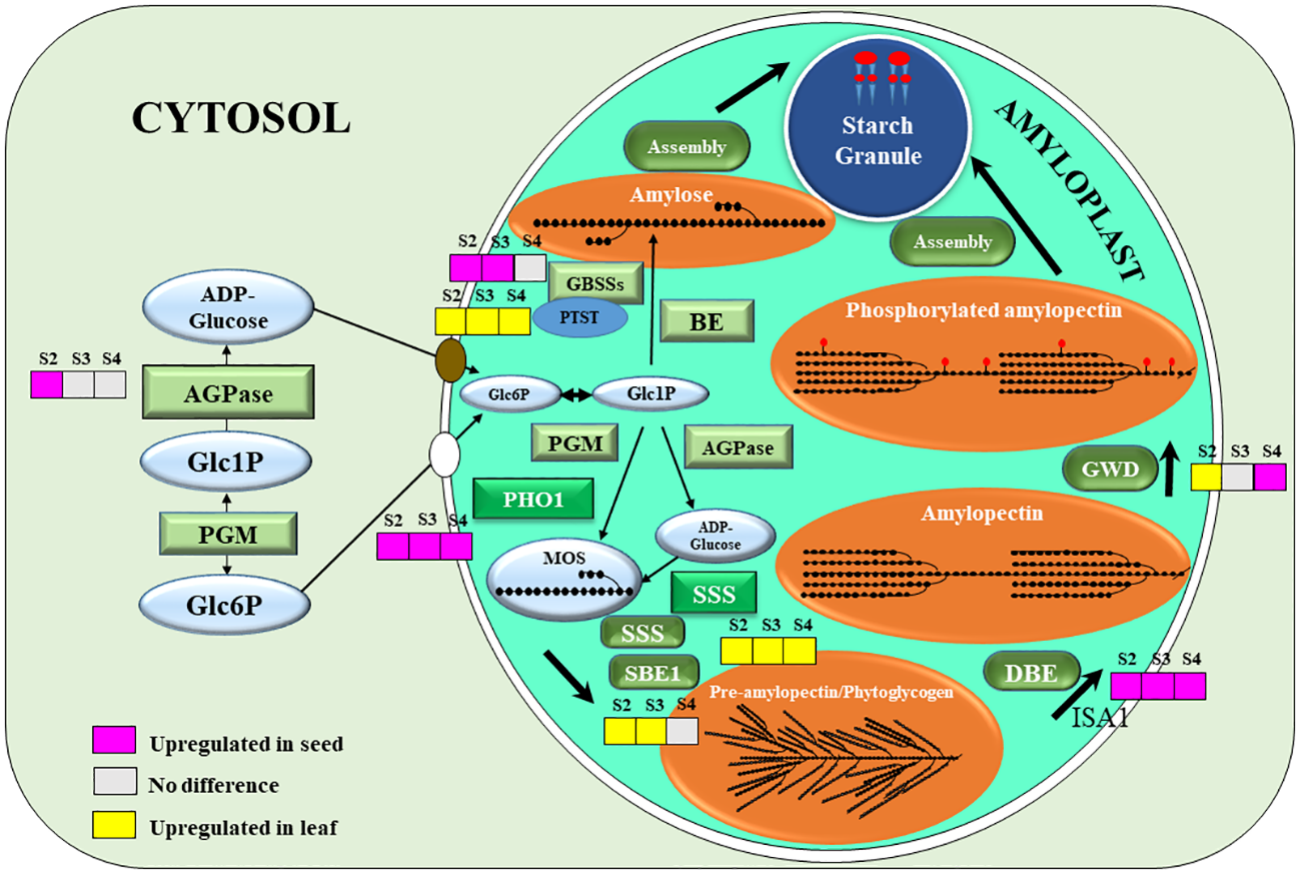
A schematic representation of starch biosynthesis in seeds: grey colour (no significantly difference), pink colour (the gene expression was upregulated in seeds), and yellow colour (the gene expression was upregulated in leaf). The crucial starch biosynthesis-related genes that were significant difference at p < 0.05, including ADP-glucose pyrophosphorylase (AGPase), granule-bound starch synthase 1 (GBSS1), granule-bound starch synthase 2 (GBSS2), protein targeting to starch (PTST), phosphoglucomutase (PGM), α-1,4 glucan phosphorylase L isozyme 1 (PHO1), F) soluble starch synthase (SSS), starch branching enzyme 1 (SBE1), starch debranching enzyme (DBE), isoamylase 1 (ISA1), α-glucan water dikinase (GWD). The precursors and intermediates of the pathways are glucose-6-phosphate (Glc6P), glucose-1-phosphate (Glc1P), malto-oligosaccharide (MOS).

Genes encoding key and crucial enzymes of the starch biosynthesis pathway were plotted into the diagram representing the sub-cellular localization and the DEGs of each gene. ADP-glucose pyrophosphorylase (AGPase), the key first committed step enzyme of starch biosynthesis, was only up-regulated in the stage 2 (**Figure 4**). Granule-bound starch synthases (GBSSs), which is the main amylose biosynthetic enzyme, were up-regulated in stage 2 and 3 (**Figure 4**). These 2 enzymes are initially located as soluble enzymes? in the stroma. However, Protein Targeting to Starch (PTST), which has a crucial role in delivery of GBSS to the starch granule surface was down-regulated in seeds (**Figure 4**). Plastidial starch phosphorylase 1 (PHO1), the major phosphorylase in the amyloplast stroma, was also up-regulated in all 3 developing stages of seeds. However, soluble starch synthase (SSS) was down-regulated in all stages, and the starch-branching enzyme 1 (SBE1) was down-regulated in stage 2 and 3 only. Isoamylase 1 (ISA1), which is involved in debranching of the pre-amylopectin during biosynthesis, was up-regulated in all 3 stages of seed development. Glucan water-dikinase 1 (GWD1), the starch phosphorylator mainly involved in amylase mediated starch mobilization (Blennow and Engelsen, 2010), but also starch biosynthesis (Xu et al., 2017) gradually increased from stage 2 to stage 4 (**Figure 4**).

**Figure 5:**
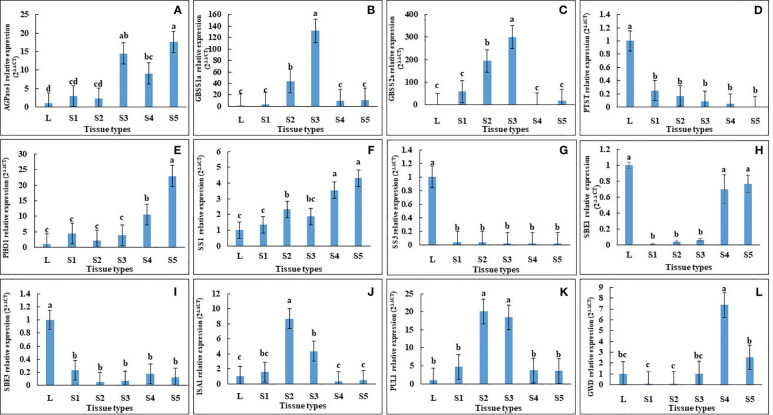
Relative expression analysis of starch biosynthesis genes in leaf and seeds at different developmental stages of *Vigna radiata* cv. CN84-1. The starch biosynthesis-related genes that were differentially expressed, including **(A)** ADP-glucose pyrophosphorylase 1 (AGPase1), **(B)** granule-bound starch synthase 1 (GBSS1), **(C)** granule-bound starch synthase 2a (GBSS2a), **(D)** protein targeting to starch (PTST), **(E)** α-1,4 glucan phosphorylase L isozyme 1 (PHO1), **(F)** soluble starch synthase 1 (SS1), **(G)** soluble starch synthase 3 (SS3), **(H)** starch branching enzyme 1 (SBE1), **(I)** starch branching enzyme 3 (SBE3), **(J)** isoamylase 1 (ISA1), **(K)** Pullulanase 1(PUL1), **(L)** α-glucan water dikinase (GWD). Different letter denotes significant difference at p < 0.05.

In agreement with this, qRT-PCR of AGPase, PHO1 and SS1 showed a gradual increase along the developmental stages of seeds, with the lowest expression in leaves (**Figure 5**). In contrast, PTST1 and SBE3 were down-regulated in all seed developmental stages, while ISA1, GBSS1, GBSS2, PUL and GWD1 were up-regulated only in seed stage 2 and stage 3 (**Figure 5**).

### 3.4 Other pathways related to the regulation and metabolism of seeds

**Figure 6:**
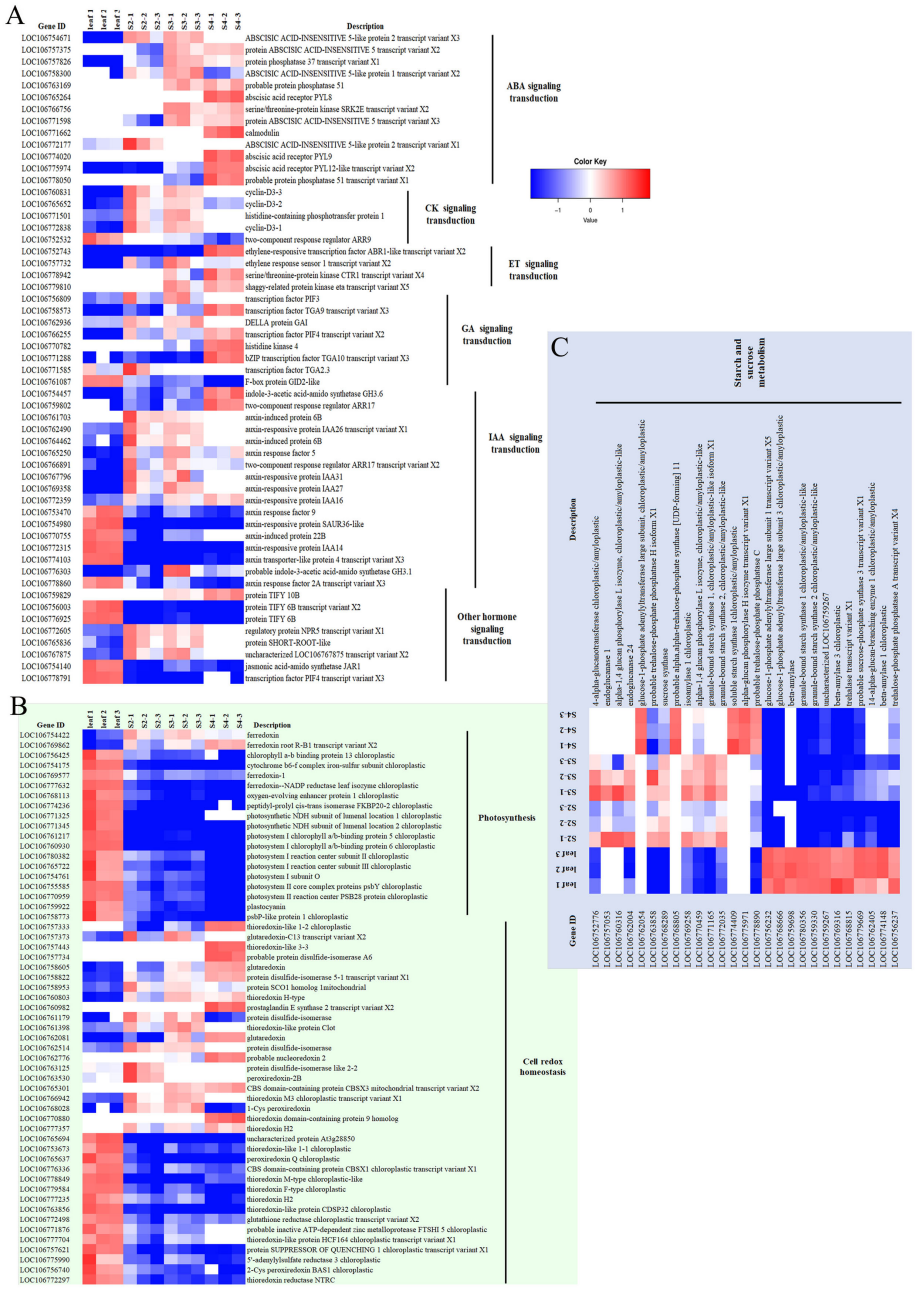
The expression patterns of DEGs of leaf and seed at different stages are focused and displayed as the log2fold change (P_adj_ < 0.05) of the DEGs. **(A)** The DEGs in the group of hormone signaling transduction, **(B)** The DEGs in the group of photosynthesis and cell redox homeostasis, **(C)** The DEGs in the group of starch and sugar metabolism. The colour scale bars indicate normalized expression levels of DEGs from leaf replication 1-3 (Leaf 1-3), seed stage 2 replication 1-3 (S2-1-3), seed stage 3 replication 1-3 (S3-1-3), and seed stage 4 replication 1-3 (S2-1-3). The heat map was constructed by Microsoft Excel 365.

Based on KEGG and Gene Ontology (GO) annotation, 4 pathways, that have been reported to be involved in seed development, were selected i) Plant hormone signal transduction (ko04075), ii) Photosynthesis (ko00195), iii) Cell redox homeostasis (GO:0045454), and iv) Starch and sucrose metabolism (ko00500); as shown in **Figure 6**.

Hormone signal transduction was one of the largest groups of DEGs that were up-regulated during seed development. The four most abundant members of this cascade included abscisic acid (ABA), ethylene (ET), auxins (IAA), cytokinins (CK) and gibberellic acid (GA)- signaling transduction. Most of the members of these hormone signaling components were gradually up-regulated with the lowest expression in leaf and highest expression in stage-4-seeds, including protein ABSCISIC ACID-INSENSITIVE 5, protein phosphatase 2C 37, abscisic acid receptor PYL8, PYL9, PYL12-like in ABA signaling transduction; ethylene-responsive transcription factor ABR1-like, ethylene response sensor 1, serine/threonine-protein kinase CTR1, shaggy-related protein kinase transcription factor PIF3 of the ET signaling transduction pathway. Transcription factor TGA9, PIF4, bZIP transcription factor TGA10 and histidine kinase 4 from GA signaling transduction; indole-3-acetic acid-amido synthetase GH3.6, two-component response regulator ARR17, and auxin-responsive protein IAA16 from IAA signaling transduction also illustrated the increasing role of indole-3-acetic acid (IAA) in the late-developmental stage (stage 4) of mung bean seeds (**Figure 6A**). The second pattern of DEGs was genes that were highly expressed during the middle stage of seed development (stage 2 and 3), and then decreased in stage 4. These included e.g. cyclin-D3-3 and cyclin-D3-2 from CK signaling transduction pathway; DELLA protein GAI, auxin-induced protein 6B, auxin-responsive protein IAA26 from GA and IAA signaling transduction (**Figure 6A**). On the other hand, F-box protein GID2-like and auxin response factor 2A gradually decreased their expression during seed development (**Figure 6A**).

Most of photosynthesis-related DEGs were highly regulated in leaf over developing seeds with the exception of ferredoxin root R-B1 that gradually increased the expression during seed development, while ferredoxin (**Figure 6B**), was highly expressed during the middle stage of seed development (stage 2 and 3). A number of photosynthesis-related DEGs showed the highest expression in leaf and continuously decreased across seed developmental stages, including chlorophyll a-b binding protein, cytochrome b6-f complex iron-sulfur, ferredoxin-A, oxygen-evolving enhancer protein 1-3, photosystem I reaction center subunit, photosystem II reaction center and plastocyanin (**Figure 6B**).

Cell redox homeostasis also possessed all 3 groups of the expression patterns. Certain DEGs such as glutaredoxin, protein disulfide-isomerase 5-1, CBS domain-containing protein CBSX3 mitochondrial, thioredoxin domain-containing protein 9 homolog and thioredoxin H2 continuously increased the upregulation in seeds across the developmental stages as compared with the low leaf expression level (**Figure 6B**). The second group were protein disulfide-isomerase, thioredoxin-like protein Clot, protein disulfide-isomerase, protein disulfide-isomerase like 2-2, peroxiredoxin-2B, thioredoxin M3 and 1-Cys peroxiredoxin, which were upregulated in seed stage 2 and 3 (**Figure 6B).** Additionally, thioredoxin F-type protein SUPPRESSOR OF QUENCHING 1 and 5’-adenylylsulfate reductase 3 decreased continuously across seed developmental stages (**Figure 6B**).

Most components of the starch and sucrose metabolism were more highly expressed in leaves than in developing seeds. However, glucose-1-phosphate adenylyltransferase large subunit; probable α,α-trehalose-phosphate synthase [UDP-forming] 1; soluble starch synthase 1; α-glucan phosphorylase H isozyme were gradually upregulated across seed developmental stages (**Figure 6C**). On the other hand, 4-α-glucanotransferase, endoglucanase 1, α-1,4 glucan phosphorylase L isozyme, endoglucanase 24, putative trehalose-phosphate phosphatase H, sucrose synthase, isoamylase 1, α-1,4 glucan phosphorylase L isozyme, granule-bound starch synthase 1 and 2 were upregulated only in the developmental stages 2 and 3 (**Figure 6C**). Other isoforms of the same genes also showed up-regulation in the leaf-upregulated group (**Figure 6C**).

### 3.5 Microscopic analyses of starch granules

**Figure 7:**
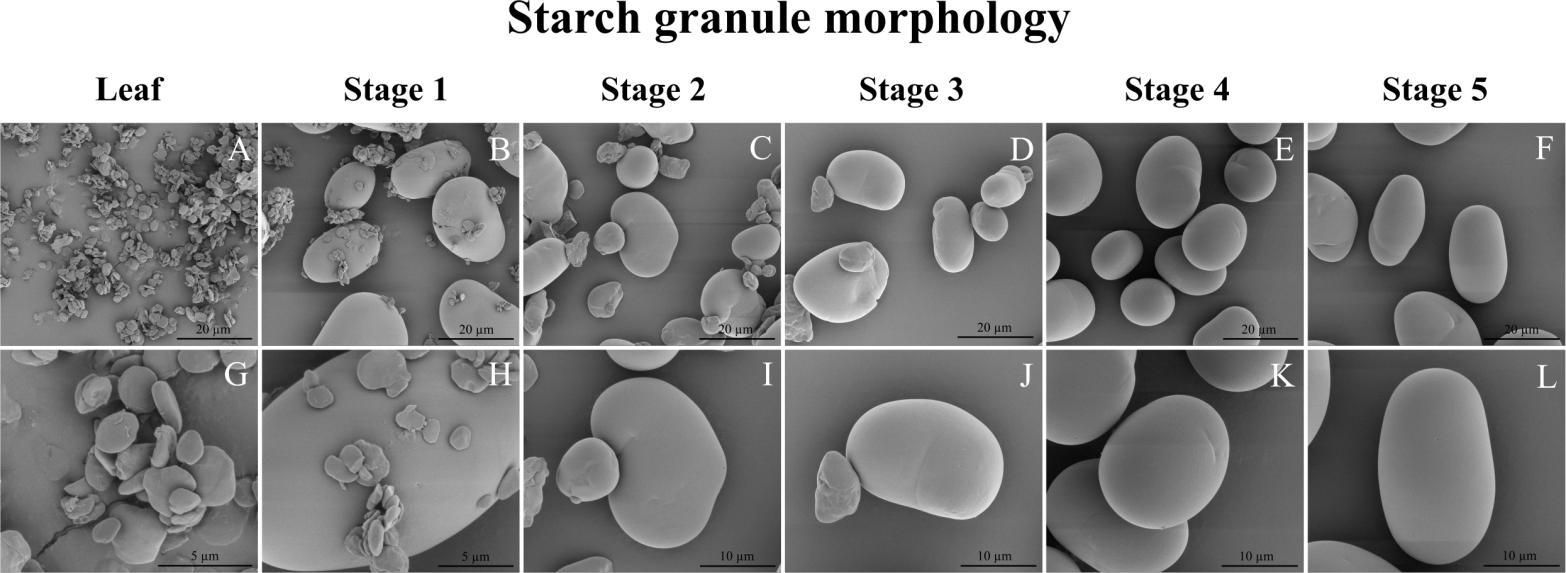
Scanning electron micrographs of isolated mung bean starch granules. **(A–F)** Starch granules at 5000x magnification. **(G)** Starch granules at 25,000x magnification. **(H)** Starch granules at 20,000x magnification. **(I–L)** Starch granules at 10,000x magnification. **(A, G)** Leaf starch; **(B, H)** Seed stage 1(5-6 DAF); **(C, I)** Seed stage 2 (10-11 DAF); **(D, J)** Seed stage 3 (14-15 DAF); **(E, K)** Seed stage 4 (17-18 DAF); Seed stage 5 (24-25 DAF).

The starch granules found in leaves were notably smaller in size compared to those observed at all stages of seed development. Leaf starch granules exhibited a distinct flat and angular morphology, resembling those of typical transitory starch granules in e.g. Arabidopsis (e.g. Zeeman et al., 2002), whereas the starch granules in seeds had a smoother contour and an oval-like shape (**Figure 7**). In particular, the starch granules in stage 1 seeds displayed a combination of flat-shaped granules akin to those in leaves and oval/kidney-like granules similar to those in mature seed starch granules (**Figures 7, B, H**). Seed starch granules in stages 2 and 3 were characterized by a mixture of small oval/kidney-like granules with diameters ranging from approximately 7 to 11 µm and larger oval/kidney-like granules measuring around 16 to 25 µm in diameter (**Figures 7, B, H**). However, it’s worth noting that the small oval/kidney-like granules disappeared after reaching 16 days after flowering (DAF), specifically in stage 4 and stage 5 seed.

### 3.6 Molecular size distribution

**Figure 8:**
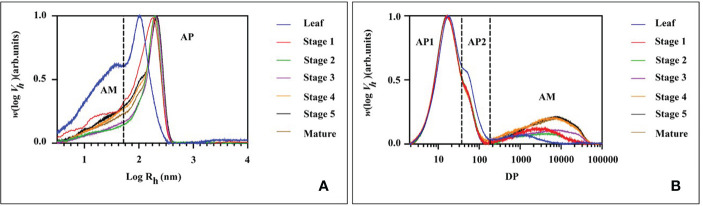
SEC weight distributions of **(A)** native (fully branched) starch molecules and **(B)** chain length distribution of debranched starch molecules from mung bean leaf and different stage of seed starches.

The molecular size distribution of the starch was determined at various stages of endosperm development and in leaf starch using size-exclusion chromatography (SEC, **Figure 8** and **Table 1** for the statistical analysis). Across all tested samples, two distinct peaks were consistently observed in the weight distribution. These peaks were classified into two categories: low molecular weight (LM, with hydrodynamic radius, Rh, ranging from 1 to 60 nm) typical for amylose and high molecular weight (HM, with Rh > 60 nm) typical for amylopectin (**Figure 8A**). It is noteworthy that the molecular size distribution of the starch in transient leaf starch markedly differed from that of storage starch. Leaf starch exhibited a combination of smaller LM and HM sections when compared to starches found in seeds. Moreover, leaf starch contained a higher proportion of LM components. During the growth from stage 1 to stage 3, both LM and HM exhibited simultaneous increases in Rh. However, from stage 3 to stage 4, there was a notable reduction in the RC of HM, accompanied by an increase in relative content of LM, with no significant alterations observed until maturity.

**Table 1:** Relative contents (RC) and average chain lengths (ACL) of debranched amylopectin (AP) and amylose (AM) fractions as deduced from Gel Permeation Chromatography (GPC) data.

Sample	RC_de-AP1_ (%)	RC_de-AP2_ (%)	RC_de-AM_ (%)	ACL _de-AP1_	ACL _de-AP2_	ACL _de-AM_	Rh_na-LM_ (nm)	Rh_na-HM_ (nm)	RC_na-LM_ (%)	RC_na-HM_ (%)
Leaf	65.8 ± 0.3b	16.3 ± 0.1bc	17.9 ± 0.2b	9.6 ± 0.0b	49.6 ± 0.4bc	2254.4 ± 59.7b	11.7 ± 0.2b	91.4 ± 2.8d	24.3 ± 0.9ab	75.7 ± 0.9ab
Stage 1	69.7 ± 0.0a	14.5 ± 0.2c	15.8 ± 0.2c	9.7 ± 0.0b	48.3 ± 0.3c	2210.4 ± 55.3b	9.7 ± 0.3b	119.6 ± 1.0c	20.3 ± 2.8b	79.7 ± 2.8a
Stage 2	68.0 ± 0.2a	18.7 ± 0.6a	13.3 ± 0.4c	12.1 ± 0.4a	50.8 ± 0.2ab	2215.9 ± 190.5b	13.5 ± 1.3a	160.3 ± 1.4a	21.3 ± 2.7b	78.7 ± 2.7a
Stage 3	65.6 ± 0.8a	16.5 ± 0.7ab	17.9 ± 0.1b	12.4 ± 0.1a	48.9 ± 0.8b	3679.2 ± 64.7a	17.5 ± 2.4a	161.8 ± 0.9a	21.2 ± 0.8b	78.8 ± 0.8a
Stage 4	55.9 ± 0.3b	15.1 ± 0.2b	29.0 ± 0.1a	11.9 ± 0.3a	52.3 ± 0.3a	3072.3 ± 75.3a	17.0 ± 1.9a	151.9 ± 2.2a	27.3 ± 0.2a	72.7 ± 0.2b
Stage 5	57.0 ± 0.4b	14.8 ± 0.1b	28.2 ± 0.5a	12.2 ± 0.5a	50.3 ± 1.0ab	3597.7 ± 40.9a	13.2 ± 1.2a	153.1 ± 4.7a	28.0 ± 0.8a	72.0 ± 0.8b
Mature	56.0 ± 2.3b	15.9 ± 1.0b	28.1 ± 1.3a	12.7 ± 1.3a	51.0 ± 0.6ab	3578.7 ± 85.5a	17.4 ± 1.5a	149.1 ± 4.1a	27.3 ± 1.4a	72.7 ± 1.4b

RC_de-X_: relative amount of fraction X of debranched sample (AP1: DP 6-36; AP2: DP37-170; AM: DP171-100000).

ACL_de-X_: the average chain length (DP) of the fraction X of debranched sample.

Rh_na-LM_: hydrodynamic radius of low molecular weight fraction (LM: Rh<60 nm) of native sample.

RC_na-LM_: relative amount of low molecular weight (LM: Rh<60 nm) fraction of native sample.

Rh_na-HM_: hydrodynamic radius of high molecular weight fraction (HM: Rh>60 nm) of native sample.

RC_na-HM_: relative amount of high molecular weight (HM: Rh>60 nm) fraction of native sample.

Values with different letters in the same column are significantly different at p < 0.05, n=3.

Further analysis of the starch fraction involved the determination of chain length distribution following treatment with debranching enzymes. This permits discrimination of chains derived from amylopectin (shorter chains) and amylose (longer chains). Hence, the chains were categorized based on their degree of polymerization (DP) into three distinct groups: short amylopectin (AP1, degree of polymerization (DP) 6–32), long amylopectin (AP2, DP 33–100), and amylose (DP > 100) (**Figure 8B**). Comparatively, leaf starch exhibited shorter AP1 and amylose chains in comparison to storage (seed) starch, a characteristic also found for potato (Blennow and Engelsen, 2010). For the seed starch, the average chain length (ACL) of AP1 increased from stage 1 to stage 2. After stage 2, there was a decrease in AP content, accompanied by a prolongation of amylose chains and an increase in their relative content. Interestingly, there were no significant differences in the molecular structures of seed starch observed after stage 3 and through maturity.

## 4 Discussion

### 4.1 Metabolic alteration during mung bean seed development

Mung bean is one of the legume seeds whose starch has been utilized in the food industry due to its transparency, high viscosity properties, and high nutritional values (Huong et al., 2021). However, the molecular regulation of the mung bean seed development versus leaf in still far from being fully understood. Transcriptomic analysis is an important tool for e.g. enabling improvement of seed quality in future heathy food industries (Zhang et al., 2015). The present study is a comparative investigation of starch biosynthesis, related-regulatory mechanisms, and basic starch properties in developing mung bean seeds and leaves.

The number of DEGs that were up- and down-regulated in seeds were similar; however, the number of DEGs that were down-regulated in all 3 stages of the developing seeds were more common (**Figure 2**). These DEGs were in the photosynthesis, plant hormone signaling, starch and sucrose metabolism (**Figure 3B**). In the context of seed development, it is noted that green seeds can perform photosynthesis; however, this process is usually insufficient for the production of storage compounds, embryo development, and energy generation. Most of the photosynthetic products are transported from the source leaves (Watson and Duffus, 1988; Galili et al., 2014). Seed photosynthesis contributes to overall energy provision and helps counter the oxygen and energy deficit caused by high biosynthetic activity in seeds. During seed development, seeds have increased oxygen requirements for respiration Celdran et al. (2015) and Kaladharan and Vivekanandan (1990) also found a remarkable 4-fold increase in the rate of oxygen release in chloroembryos of cluster bean (*Cyamopsis tetragonoloba*) compared to leaf chloroplasts, and a positive correlation between seed oxygen demand and photosynthesis. Our findings supported this (**Figure 3A**), since the oxidative phosphorylation pathway was highly regulated in seeds as compared to leaves. Redox signals are suggested to be facilitated by the ferredoxin/thioredoxin cascade (**Figure 6B**) as demonstrated by the up-regulation in seeds of several ferredoxins and thioredoxins with the highest expression in stage 4 seeds in both photosynthesis and cell redox homeostasis groups. Redox signals have been reported to influence the activity of specific biosynthetic enzymes such as NADPH oxidase, fructose bisphosphatase and NADP-malate dehydrogenase, allowing the plastidial redox pool to participate in signal transmission between the plastid and nucleus (Buchanan and Balmer, 2005; Tschiersch et al., 2011). Thioredoxin has been reported to accumulate in wheat nucleus during oxidative stress and certain family of thioredoxin plays a role in transcription factor regulation in plant transcription controls (Buchanan and Balmer, 2005). Especially, redox regulation is fundamentally linked to starch biosynthesis by a thioredoxin-mediated general activation of a number of starch biosynthetic enzymes (Glaring et al., 2012; Skryhan et al., 2018) e.g. starch synthase I (Skryhan et al., 2015) and also regulates the partitioning of the GWD1 onto the starch granule surface during night (Mikkelsen et al., 2005).

The translocation of photosynthases is also regulated by plant hormones. ABA promotes phloem unloading, while IAA promotes phloem loading, phloem transport and activity of sinks (Brenner and Cheikh, 1995). GAs was maximally produced during the period of rapid seed growth (between stage 2-4 in our experiment, **Figure 1**), but significantly decreased at the early seed development and in the pea seed maturation stage (stage 1 and 5, respectively) (Sponsel, 1983). The distribution of ABA within plants varies throughout the day and across different stages of plant development. Notably, in developing soybean plants, significant ABA accumulation occurs in the seeds during mid-pod filling. This stage is pivotal, and removing the developing pods at this time causes ABA accumulation within the leaves. This suggests that, during mid-pod fill, the developing seeds act as the primary sites for ABA storage, with other developmental stages have multiple sites contributing to ABA accumulation (Hein et al., 1984; Ali et al., 2022). The seeds are also able to synthesize ABA *de novo*, except in some plants or cultivars that acquire most of their ABA from leaves. ABA accumulated during seed maturity also contributes to seed vigor (Montrichard et al., 2020). This observation may explain the elevated expression of ABA receptors, including PYL8, PYL9, and PYL12, during stages 3 and 4 of seed development (**Figure 6A**). Furthermore, the components of ABA-responsive elements, such as abscisic acid-insensitive 5, phosphatase 2C 51, and SRL2E kinase, also exhibit increased expression levels during these specific developmental stages. (**Figure 6A**).

Apart from oxidative phosphorylation and plant hormone signaling transduction, KEGG enrichment also suggested that up-regulation of DEGs was related to protein processing in endodermic reticulum, spliceosome, and ubiquitin mediated proteolysis (**Figure 3A**). In the course of seed development, the endoplasmic reticulum (ER) plays a crucial role in efficiently synthesizing and structurally maturing storage proteins within a short timeframe (Vitale et al., 2022). Several bZIP transcription factors participate in the activation, splicing, and control of the unfolded protein response (UPR), influencing various UPR-associated processes such as protein folding assistance, translational suppression, and programmed cell death, as detailed by Wang et al. (2014). In seed stage 4, an bZIP transcription factor was up-regulated over other stages and in leaves (**Figure 6A**). It has been reported that bZIP interacts with BiP, a crucial factor facilitating the controlled entry of newly synthesized polypeptides into the endoplasmic reticulum (ER), thereby preventing uncontrolled protein aggregation (Srivastava et al., 2013). UPR employs various protective mechanisms to eliminate permanently misfolded secretory proteins. One of these mechanisms is ERAD (endoplasmic reticulum-associated degradation), which e.g. serves as the initial line of defense. This process involves the extraction of faulty polypeptides from the ER to the cytosol, followed by their targeting for degradation through ubiquitin-mediated proteasomal pathways (van Anken et al., 2021).

Glyoxylate and dicarboxylate metabolism was also highly down-regulated in seeds (**Figure 3B**). This DEGs cluster was mainly explained by the central metabolic pathway of the cells, including pathways that provides precursors of glycolysis, pyruvate biosynthesis, citrate cycle and amino acid biosynthesis. It is noteworthy that seeds are the primary sink organ, most of the precursors of both primary and secondary metabolites are mainly synthesized in leaves, and then transported to the seeds for growth and development of the embryo and storage compartments (Gallardo et al., 2008). Therefore, the glyoxylate and dicarboxylate metabolism displayed more pronounced expression in leaves.

### 4.2 Starch biosynthesis alteration during mung bean seed development

In this study we identified and investigated differentially expressed genes (DEGs) with function in starch biosynthetic pathways (synthesis and mobilization) in mung bean leaves and developing seeds. AGPase, GBSS1a, GBSS2a, and ISA1 were upregulated in seeds, suggesting that the limiting steps of starch biosynthesis in seeds might not be associated with these genes. Conversely, PTST, PGM, and SBE1 were downregulated in seeds, indicating their potential as targets for overexpression aiming to increase starch accumulation in seeds of mung bean and related species.

The findings obtained from the transcriptomic analysis were validated using quantitative real-time polymerase chain reaction (qRT-PCR). The expression levels of several key genes, including PTST, ISA1, PHO1, GBSS1a, GBSS2a, PUL, SS1, SS3, and GWD, were consistent with the transcriptomic data, confirming the reliability of the transcriptome data (**Figures 4**, **5**). However, AGPase1 exhibited divergent expression patterns compared to the transcriptomic analysis (**Figures 4**, **5**).

A developmental stage-wise examination revealed intriguing expression trends. AGPase1, a key enzyme in starch biosynthesis, displayed a gradual increase across seed developmental stages, implying increased starch production throughout development. The increasing expression of PHO1, known for its role in starch granule initiation and maturation, suggested a corresponding increase in starch granule content or accumulation (Hwang et al., 2016). The elevated expression of SS1, implicated in amylopectin biosynthesis, indicated increased amylopectin biosynthesis during mung bean seed development (Seung, 2020). The augmented expression of GWD pointed towards a role of starch phosphorylation for enhanced amylopectin biosynthesis over the course of seed development. The inclusion of phosphate groups in the synthesis process could potentially disrupt the semi-crystalline arrangement of the granule matrix (Blennow and Engelsen, 2010), thus aiding the functions of starch synthases and branching enzymes on the surface of the starch granules (Skeffington et al., 2014). The decrease of expression of GWD in only stage 5 may be due to the delay in amylopectin biosynthesis (**Figure 5**). SBE1 also displayed increased expression across the developmental stages but not higher than the expression in leaf. The increased expression of SBE1 may confer increased branching of pre-amylopectin.

The second expression pattern comprised the DEGs that possessed the upregulation in the middle stage of seed development. This group was consisted of GBSS1a, GBSS2a, ISA1 and PUL1. GBSS1 is a key enzyme in amylose synthesis in starch granule. The *Wx* (GBSS1-encoding gene) is, however, also responsible for the biosynthesis of amylopectin as demonstrated by GBSS1 overexpression lines on the *wx* null-mutant background, which resulted in 14.9–16.0% increase in the starch weight for the overexpressing lines, with a significant increase in extra-long unit chains (ELCs) of amylopectin (7.5–8.4% of amylopectin weight) (Hanashiro et al., 2008). In grass species, GBSS1 expression is confined to the endosperm and pollen grains, whereas GBSS2 is predominantly found in vegetative tissues and the pericarp. Conversely, in peas, GBSSa exhibits enhanced expression in embryos, while GBSSb is notably enriched in leaves, pods, and nodules (Seung, 2020). However, in mung bean, 4 GBSSs were represented in the DEGs. Two were up-regulated in seeds (GBSS1a and GBSS2a) and the other two were enriched in leaf (GBSS1b and GBSS2b) (**Supplementary Table S2**).

Although ISA1 and PUL1 exhibit distinct preferences for substrates (phytoglycogen and pullulan, respectively), they both serve as starch-debranching enzymes in amylopectin biosynthesis. Importantly, these two enzymes have the capacity to compensate for each other’s functions (Kubo et al., 1999). In contrast, bacterial isoamylase is limited in its ability to remove individual branch glucosyl units from branched maltodextrins, and its action on maltosyl units linked by α-(1,6) linkages is notably slow. However, it displays a significantly higher efficiency, approximately 7 times faster, in hydrolyzing the α-(1,6) linkages found in amylopectin compared to pullulanase (Robyt, 2009). The low isoamylase activity, that was observed in Sugary-1 mutation in rice results in exclusive accumulation of phytoglycogen instead of amylopectin in the endosperm (Kubo et al., 1999). ISA1and PUL1 were highly expressed in stage 2 and 3 seeds perhaps implying that the amylopectin was dominantly synthesized in stage 2 and 3 and then decreasing during stage 4 and 5.

### 4.3 Hormonal, photosynthesis, redox alteration in mung bean seed starch biosynthesis

As illustrated in **Figure 1**, there was a significant expansion of seeds during stages 3 and 4. This phenomenon was substantiated by the gradual upregulation of ABA signaling transduction, peaking at stage 4 (**Figure 6A**). ABA plays a role in facilitating sucrose unloading and its accumulation in soybean cotyledons (Brenner and Cheikh, 1995). Subsequently, sucrose undergoes degradation and is reutilized in the starch biosynthesis, which in our data is substantiated but the notable upregulation of the majority of the starch biosynthesis enzymes during stage 3 and 4. It is plausible that in seeds, a number of genes with function in auxin and gibberellin signaling transduction, are upregulated during these stages. It has also been reported that these phytohormones, exported from the sink tissues, are potentially factors contributing to enhanced photosynthesis in the source tissue. Notably, leaf photosynthetic genes also displayed an upregulation during seed development, (**Figure 6A**). This could be attributed to the substantial sink strength of the developing seeds. Regarding the redox regulation of starch biosynthesis, thioredoxin (LOC106757333) was highly expressed in seed stage 4 (**Figure 6B**), which coincided with the increasing expression of the GWD1 and SS1 (**Figure 5**). Normally, thioredoxin f (Trx*f*), thioredoxin m (Trx*m*) and NADP-dependent thioredoxin reductase C (NTRC) have been shown to induce reduction and conformational change of GWD1 and SS1, leading to the increased affinity to starch (Skryhan et al., 2018).

### 4.4 Starch morphology and properties in mung bean developing seeds and leaves

Global expression patterns during seed development can be related to very specific structural motifs in the starch as has recently been demonstrated for wheat (Zhong et al., 2023). In our study, as evident from iodide-stained cross-sections, of the mung bean seeds in stage 1 and 2, a distinct dark blue staining was clearly observed in the seed coat (**Figure 1D**). This observation coincided with the presence of flat-shaped starch granules in stages 1 to 3 of seed development (**Figure 7**).This suggests a potential connection between the flat-shaped starch granules and the starch accumulation occurring in the seed coat of mung bean seeds. The absence of flat-shaped starch granules could be attributed to a decline in photosynthesis within the seed coat, coupled with the degradation of transient storage compounds in both the pod and seed coat. These compounds are subsequently mobilized and transported to storage tissues, such as the cotyledon and embryo, as also reported in young seed coats of *Vicia faba* and peas (Weber et al., 2005; Tschiersch et al., 2011). Furthermore, sucrose synthase activity in the seed coat is closely linked to starch synthesis within the embryo (Dejardin et al., 1997), which coincided with the peak expression of sucrose synthase (LOC106768289) in stage 2 and 3 of seed development (**Figure 6C**). Additionally, the mung bean seed starch granules (**Figure 7**) were consistent with those described by Hoover et al. (1997). These starch granules exhibited irregular shapes, ranging from oval to round to bean-shaped, and displayed a wide range of sizes, spanning from 7.1 to 26.0 µm. The sizes of mung bean starch granules were smaller than the reported sizes (12-48 µm) for starches found in other legumes. Additionally, the surfaces of these mung bean starch granules appeared smooth and showed no signs of fissures (Hoover and Sosulski, 1991).

When compared with developing seed starch, leaf starch exhibited distinctive features, including a higher relative quantity and longer average chain length of long amylopectin (AP2), a reduced amount of amylose, and a more pronounced presence of low molecular weight (LW) fractions, (**Figure 8B**). These characteristics are indicative of transient starch, and this outcome may be attributed to the low expression of SS1 in leaves (**Figure 5F**). Evidencing in the mutants and transgenic plants lacking SS1 activity, these alterations tend to result in deficiencies in shorter glucan chain lengths with a degree of polymerization (DP) 6–12 (Tetlow, 2011). The suggested role of SS1 in the synthesis of shorter chain glucans is further supported by the observed increase in both the quantity and average chain length of amylose as growth progresses (**Figure 8B**). In contrast, research on waxy corn starch has indicated a slight decrease in amylose content during development (Zheng et al., 2021). Meanwhile, studies on wheat starch have revealed periodic fluctuations in both the amylose content with high degree of polymerization (DP>100) and the content of short amylopectin chains with DP < 32 during wheat grain development (Zhong et al., 2023). These variations imply that the patterns of changes in amylose and amylopectin chain lengths are dependent upon the specific plant species under investigation.

The molecular size distribution of mung bean starch and the chain length distribution of these throughout endosperm development indicated that:

1. During the transition from stage 1 to stage 2, branching seems to substantially influence the molecular size of both starch molecules, resulting in a simultaneous increase in hydrodynamic radius (Rh) of both the LM (low molecular weight fraction, Rh<60 nm) and the HM (high molecular weight fraction, Rh>60 nm). In this phase, starch biosynthesis is primarily associated with a notable augmentation in the average lengths of amylopectin chains, particularly the AP1 fraction (DP 6-32). This observation suggests active biosynthesis and elongation of amylopectin chains during this developmental stage, which corresponded with the increased expression of SS1, ISA1 and PUL at stage 2 (**Figures 5F, J, K**).
2. In stage 2, a shift in the composition of starch molecules was observed. The content and length of amylose chains increased, while the synthesis of amylopectin was limited during this period, as supported by the augmentation of the GBSS1 and GBSS2a expression in stage 2 and 3 of seed development (**Figures 5B, C**). Consequently, the relative content of amylose increased, whereas that of amylopectin decreased. The expression of ISA1 and PUL also peaked in seed stage 2 and started decreasing at stage 4 or 5, together with the peak of GWD1 in seed stage 4 and sharply drop at stage 5. This might lead to the observed Rh of the HM starch molecules and the average chain length of amylopectin (ACLAP) remained nearly unchanged. This suggests that there was no substantial “trimming” of amylopectin molecules during developmental stage.
3. After stage 4, a relative stability in starch structure was observed, with no significant changes noted until maturity was reached. This phase suggests that the essential molecular architecture of mung bean starch remains relatively consistent during late developmental stages and into maturity. Studies on wheat starch have revealed periodic fluctuations, including an increase in amylopectin content and a decrease in amylose content in the late stage (Zhong et al., 2023). However, these fluctuations were not observed in our study. This difference underscores the influence of the botanical source on starch composition and developmental dynamics.

In conclusion, significant differences have been observed in the expression of starch biosynthesis genes, as well as in other regulatory systems and the morphology of starch granules, between mung bean seeds and leaves. The transition from transient starch (characterized by flat-shaped granules) to storage starch (with oval and kidney-shaped granules) is evident from stages 1 to 3. However, from stage 4 onwards, no alterations in starch morphology and properties are observed. This suggests that modifications in starch properties should be targeted towards the highly differentiated differentially expressed genes (DEGs) observed in stages 2 to 4 to minimize the effects on growth. Nevertheless, these postulations require further studies to confirm. The data obtained from this study can also serve as a valuable toolkit for future endeavors in genetic engineering and molecular breeding, with the aim of enhancing the amylose content of mung beans or tailoring the unique properties of mung bean starch for applications in the food and other industries.

## Data availability statement

The datasets presented in this study can be found in online repositories. The names of the repository/repositories and accession number(s) can be found below: https://www.ncbi.nlm.nih.gov/, PRJNA1033708.

## Author contributions

KU: Conceptualization, Data curation, Formal analysis, Funding acquisition, Investigation, Methodology, Project administration, Supervision, Validation, Writing – original draft, Writing – review & editing. PB: Data curation, Methodology, Validation, Writing – original draft. RS: Data curation, Investigation, Project administration, Writing – original draft. ST: Data curation, Formal analysis, Funding acquisition, Writing – original draft, Writing – review & editing. YT: Data curation, Formal analysis, Methodology, Writing – original draft, Writing – review & editing. YH: Data curation, Methodology, Writing – original draft. BP: Funding acquisition, Supervision, Writing – review & editing. AB: Conceptualization, Funding acquisition, Methodology, Supervision, Writing – review & editing.

## Supplementary material

Supplementary Table 1 Primers used in this study.

Supplementary Table 2 GBSS genes presented in DEGs.

Supplementary Figure 1 Starch content of 6 Thai mung bean cultivars.

Supplementary Figure 2 Principal component analysis chart.

Supplementary Figure 3 Differential expression volcano plot.

Supplementary Data Sheet 1 Common up- and down- regulated DEGs in all 3 stages of mung bean seed development.
