# Supplementary material for: Transcriptomics and starch biosynthesis analysis in leaves and developing seeds of mung bean provide a basis for genetic engineering of starch composition and seed quality
Source: Front Plant Sci. 2024 May 1;15:1332150. doi: 10.3389/fpls.2024.1332150 (PMC11094274; doi:10.3389/fpls.2024.1332150)
Supplement: Supplementary Table 1 — Primers used in this study. [file DataSheet_1.docx]

Supplementary Material

Transcriptomic analysis and starch biosynthesis of mung bean (*Vigna radiata*) developing seeds and leaves

Kamolchanok Umnajkitikorn, Pakpoom Boonchuen, Rattanavalee Senavongse, Sunanta Tongta, Yu Tian, Yaqi Hu, Bent Larsen Petersen, Andreas Blennow

*** Correspondence:** Corresponding Author: k.umnajkitikorn@g.sut.ac.th

# Supplementary Tables

**Table S1** Primers used in this study

| **Target Primer** | **Accession number** | **name** | **Primer sequence (5’ 3’)** |
| --- | --- | --- | --- |
| Actin-3 | XM_014658079.2 | VrActin.f | CAGTGTCTGGATTGGAGGCT |
| (LOC106771984) |  | VrActin.r | GTCCTCGACCACTTGATG |
| ADP-glucose | XM_014639830.2 | AGPase1-F | GGGCTGAGGAAAGAAAGAGCAGAG |
| pyrophosphorylase 1 (AGPase1) (LOC106757210) |  | AGPase1-R | TGGGTATGGTCCAAACAGCATGG |
| Protein Targeting to Starch (PTST) | XM_022779388.1 | PTST-F | AGGATGTCGATGCGGCACAATC |
| (LOC106758029) |  | PTST-R | CCATAACTTGCACGCTCTCAGC |
| Starch synthase1 | XM_014661395.2 | SS1-F | TTGGGTTCATTGGAAGACTGGAC |
| (SS1) (LOC106774409) |  | SS1-R | GCTTCCATGAGCTCTGGCATTG |
| Starch synthase3 (SS3) | XM_014662912.2 | SS3-F | AAGGGTGATGGAGCAAGACTGG |
| (LOC106775746) |  | SS3-R | CTTGCGTGCTGCATGGTAAAGC |
| Isomerase1 (ISA1) | XM_014654795.2 | ISA1-F | TGGACGCCAATATGTATCCCATGC |
| (LOC106769258) |  | ISA1-R | TTGATCAGGAGTGCGCAAGAGG |
| Plastidial starch phosphorylase 1(PHO1) | XM_022786882.1 | PHO1-F | GCCGCCCAAGTTTCAGTTGTATG |
| (LOC106760316) |  | PHO1-R | TAGTTGGTGGTAGGAACCGAGGAC |
| Starch-branching | XM_014646305.2 | SBE1_F | ACCATGGCATTAGGTGGAGAGG |
| enzyme1 (SBE1) (LOC106762405) |  | SBE1_R | TCCGGATGGCCAAACTCATTGC |

| **Target Primer** | **Accession number** | **name** | **Primer sequence (5’ 3’)** |
| --- | --- | --- | --- |
| Starch-branching enzyme3 (SBE3) (LOC106780166) | XM_014668421 | SBE3-F | AAGGGACCAGCCAAACAATGGG |
|  |  | SBE3-R | TTCACTGCCAGCAACGTCTAGG |
| Granule-bound  starch synthase1 (GBSS1) (LOC106771165) | XM_022784505.1 | GBSS-I_F | TTGTCACTGCCTGCCACGTTTC |
|  |  | GBSS-I_R | TGCCATTCTAATGCGGGAGGAG |
| Granule-bound | NM_001317304.1 | GBSS2a-F | AGGAGCTTGGTTTGCCTATCCG |
| starch synthase2a (GBSS2a) (LOC106759330) |  | GBSS2a-R | TCCTTTCTGTGGATCCAACCTTCC |
| Pullulanase (PUL) | XM_014658033.2 | PUL-F | ACAGGACCTTCATGGGTTTGTGG |
| (LOC106771955) |  | PUL-R | AACCCAGGAAAGCCATCGTGAC |
| Glucan water- | XM_014667687.2 | GWD_F | AAAGGGCATACTTCAGCACACG |
| dikinase (GWD) (LOC106779562) |  | GWD_R | ACCACTTCCTGAACAAGGACAGC |

**Table S2 GBSS genes presented in DEGs**

| **geneID** | **Gene name** | **length** | **Regulation in seeds** | **Log_2_FoldChange** | | | **Description** |
| --- | --- | --- | --- | --- | --- | --- | --- |
|  |  |  |  | **S2/Leaf** | **S3/Leaf** | **S4/Leaf** |  |
| gene-LOC106771165 | GBSS-Ia | 2284 | Up | 7.774483891 | 8.79114 | #N/A | granule-bound starch synthase 1 chloroplastic/amyloplastic-like |
| gene-LOC106759330 | GBSS-IIb | 2572 | Down | -5.832438208 | -5.517992 | -4.539146 | granule-bound starch synthase 2 chloroplastic/amyloplastic-like |
| gene-LOC106780356 | GBSS-Ib | 1845 | Down | -6.815039071 | -7.248496 | -6.178899 | granule-bound starch synthase 1 chloroplastic/amyloplastic-like |
| gene-LOC106772035 | GBSS-IIa | 3039 | Up | 3.465332155 | 3.12103 | #N/A | granule-bound starch synthase 2 chloroplastic/amyloplastic-like |

# Supplementary Figures


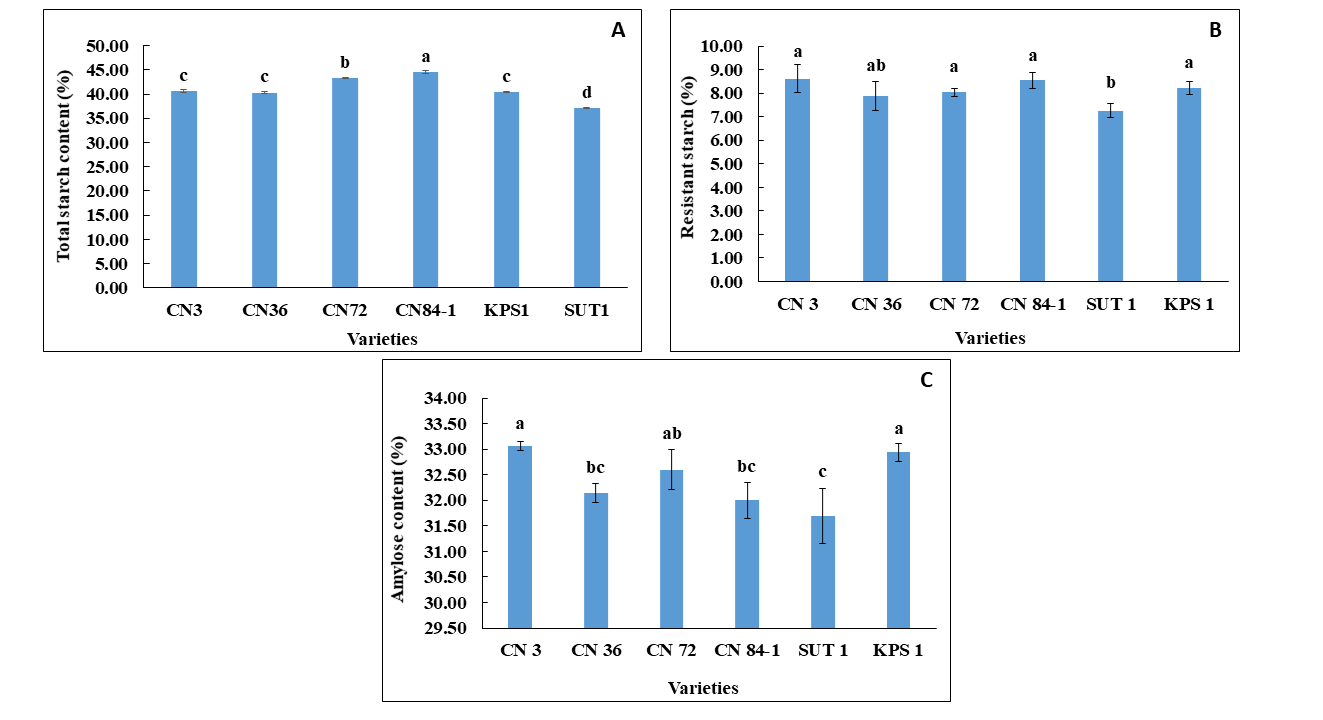


**Supplementary Figure 1.** Starch content of 6 Thai mungbean cultivars. A) Total starch (%; g starch/100 g dry seed weight); B) Amylose content (% of starch); C) Resistant starch (% of starch). The values shown are the Mean ± SE (n = 3, respectively). The different letters above the bars indicate significant differences by one-way ANOVA and Duncan’s test (*p ≤ 0.05*).


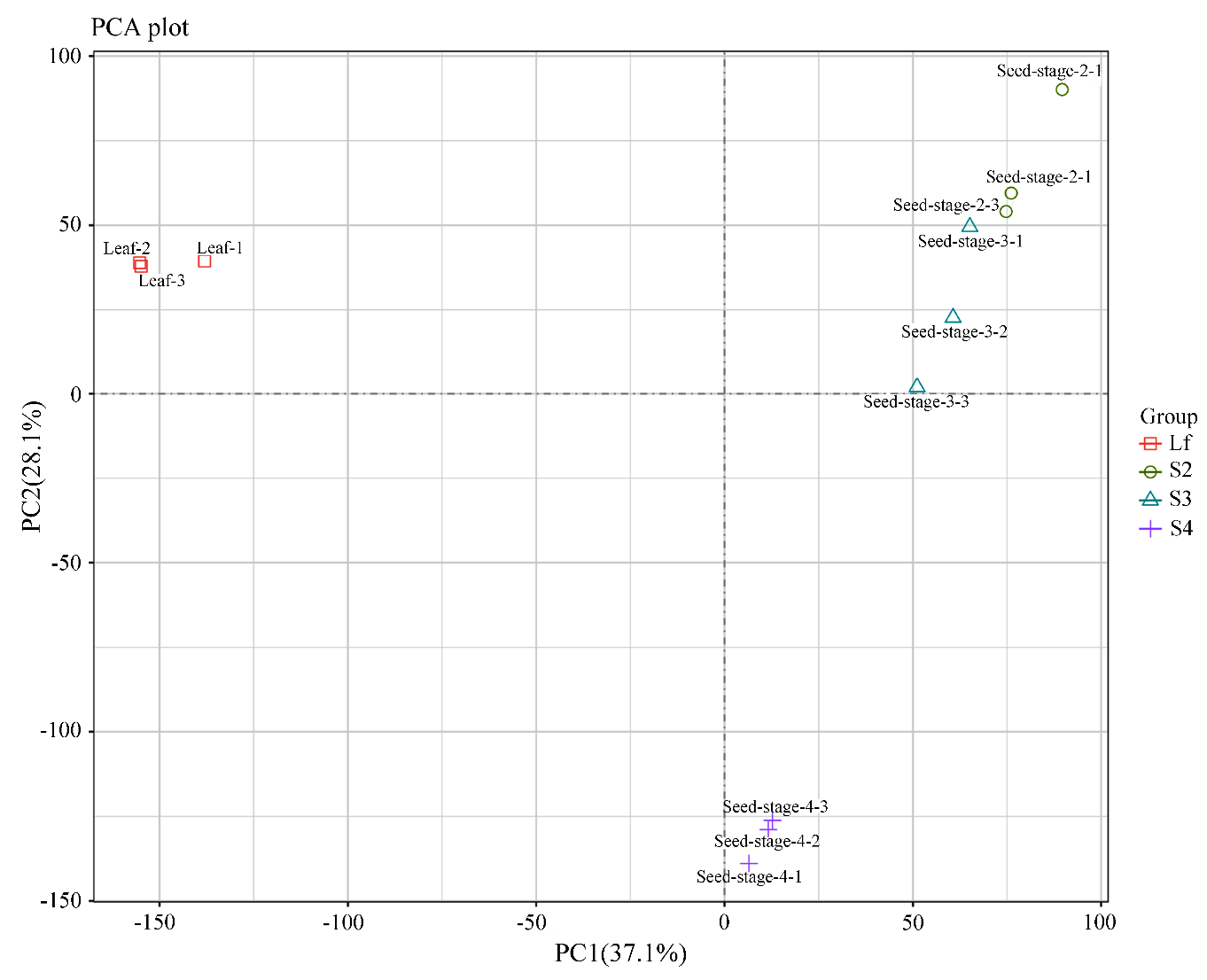


**Supplementary Figure 2.** Principal component analysis (PCA) based on the expression level of all transcripts. Sample to sample distances (within-and between-treatments) were visualized on the first two principal components. The first two axes account for 37.1% (PC1) and 28.1% (PC2) of the variation in the data. The samples were then graphically represented based on their treatment groups (seed-stages and leaf).


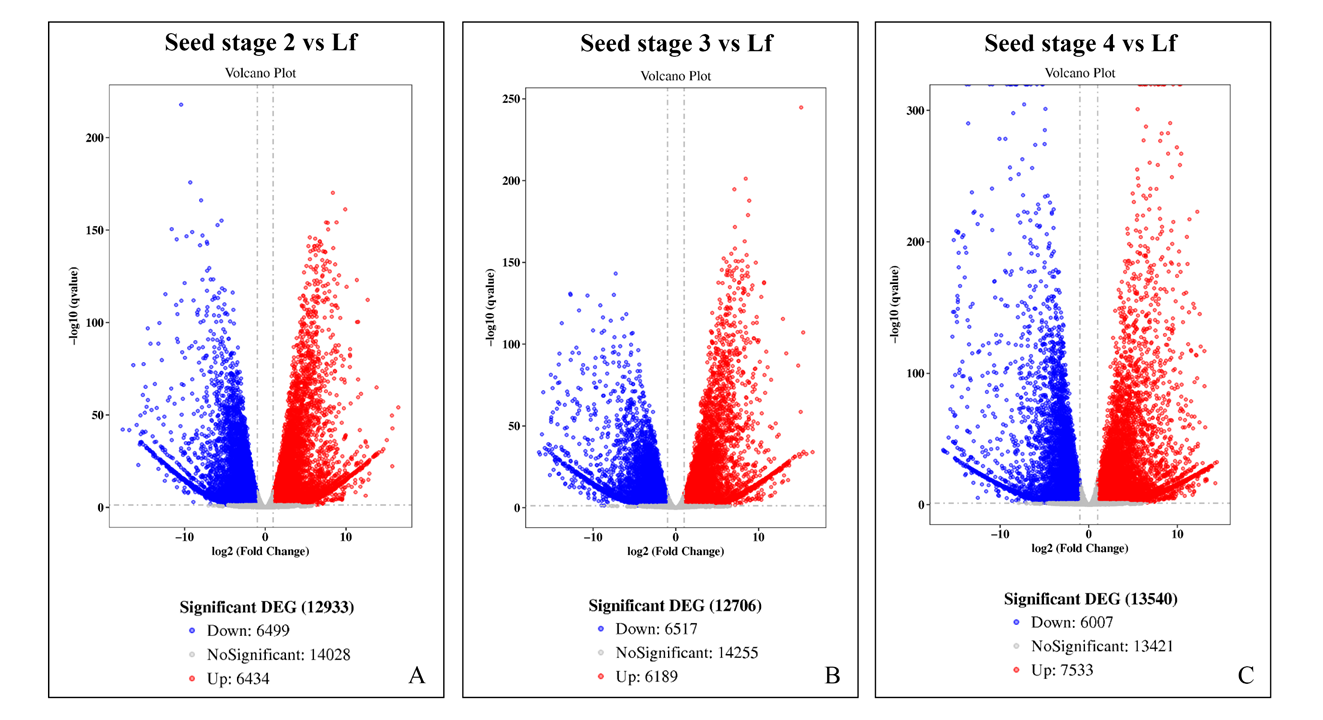


**Supplementary Figure 3.** Differential expression volcano plot, red dots represent genes that are significantly up-regulated and blue dots represent those that are significantly down-regulated. X axis: log2 fold change of gene expression. Y axis: statistical significance of the differential expression in log10 (qvalue(fdr, padj)) A) Seed stage 2 and leaf, B) Seed stage 3 and leaf, C) Seed stage 4 and leaf.
